# Specific island biogeographic and landscape features shape plant diversity and habitat specialism on edaphic quartz islands in an arid ocean

**DOI:** 10.1038/s41598-025-94562-5

**Published:** 2025-03-22

**Authors:** Pia Maria Eibes, Ute Schmiedel, Jens Oldeland, Severin David Howard Irl

**Affiliations:** 1 Biogeography and Biodiversity Lab, Institute of Physical Geography, Goethe University Frankfurt, Riedberg Campus, Altenhöferallee 1, 60438 Frankfurt am Main, Germany; 2 Organismic Botany and Mycology Lab, Institute of Plant Science and Microbiology, University of Hamburg, Hamburg, Germany; 3 Institute for Globally Distributed Open Research and Education (IGDORE), Hamburg, Germany

**Keywords:** Habitat islands, Island biogeography, Matrix vegetation, Isolation, Landscape heterogeneity, Succulent Karoo, Biodiversity, Biogeography, Biodiversity, Biogeography, Plant ecology

## Abstract

Edaphic habitat islands offer unique environmental conditions for plants and often harbour specialized floras, thus having high nature conservation value. Besides edaphic uniqueness, distinct spatial features and landscape filters characterize habitat islands. However, their role as drivers of biodiversity on habitat islands remains unclear. We aim to quantify the extent to which spatial parameters (island shape characteristics, habitat diversity) and landscape filters (insularity parameters, surrounding matrix effects) explain plant diversity on natural edaphic islands in an arid biodiversity hotspot. The quartz islands in our study area (Knersvlakte, South Africa), resemble natural edaphic islands within a semi-arid vegetation matrix, hosting unique, predominantly dwarf and locally endemic succulent plants. In a comprehensive field survey, we sampled plant diversity and richness of 47 quartz islands and analysed their spatial characteristics derived from satellite imagery. Island area and habitat diversity were the most reliable predictors of plant diversity. Including measures of landscape features, (e.g. matrix contrast) improved the predictive power of several models. However, distance-based isolation measures had little explanatory value in relation to the observed variance. These results suggest that the diversity of quartz islands can be effectively described using specific island biogeographic parameters, such as island area or habitat diversity. However, measuring isolation in edaphic island systems may require alternative quantification methods such as incorporating matrix properties. Based on the results of this study, we recommend combining different parameters in a habitat island biogeography to quantify the biodiversity of such natural terrestrial edaphic islands.

## Introduction

The foundational equilibrium theory of island biogeography (ETIB^1^)—especially with a focus on island size and isolation—was specifically developed to predict patterns of species diversity and endemism as a balancing dynamic of species’ extinction and immigration. The theory has been applied to true islands as well as habitat islands since the beginning of island biodiversity research^1,2^. The results of testing the ETIB on habitat islands and landscape fragments had, among other things, a major impact on nature conservation and landscape ecology, where habitat size, connectivity, and isolation are important factors^3^.

Habitat islands—defined here as patches with insular character located in a terrestrial environment—have indeed apparent analogies to true islands surrounded by water^4^. They represent spatially isolated, distinctive units within an adjacent contrasting matrix^4,5^. Several of these spatial parameters, particularly island area, have since been discussed to play a major role as potential predictors of biological diversity in different habitat island systems^6–9^. A representative example of this is the relevance of the species-area relationship (expressed as species-area relationship models or species accumulation curves^10,11^), which describes how species richness increases with increasing island area and appears to be stronger on true islands than on habitat islands^12,13^. Habitat diversity (commonly quantified as topographic heterogeneity) within an island often correlates with the area of the island. Habitat diversity has been identified as a further driver of higher species richness as evidenced by studies conducted on true islands^8,14,15^ and habitat islands^11,16^. Islands with higher habitat diversity probably provide more and diverse niches that translate into higher species numbers^17,18^. However, colonization, speciation and extinction on habitat islands may be affected by additional characteristics that are not addressed by the ETIB, such as the lack of knowledge of the original source of the species, interactions with the edge or matrix, and different isolation mechanisms. Therefore, several studies classified the ETIB in part as too simplistic to predict or describe the ecology of habitat islands or fragmented ecosystems^3,19–21^ or even proposed different predictors^22,23^.

While in the case of true islands, the ocean represents an inhospitable and uninhabitable barrier for most terrestrial island species, habitat islands can be delimited by a wide variety of matrix environments^4,24^. This raises the question of the extent to which the surrounding environment serves as an isolating barrier for habitat islands and illustrates the taxon-dependency of isolation in such systems^24,25^. Isolation (also referred to as insularity in the habitat island context^26^) can be measured in different aspects^15^, and—when high— usually leads to decreased species richness but increased proportions of habitat specialists on islands, which in turn would increase the relevance for conservation. In the case of habitat islands, therefore, the nature of the matrix may be a critical factor^19,24^, the importance of which has often been acknowledged in previous studies but is not easily defined or measured. Whether the matrix is an impassable obstacle for a species or not depends on many aspects. These include the dispersal ability of the species itself, how distinct the conditions of the matrix habitats are from the ones at the island habitat, as well the presence or absence of stepping stone habitats in the matrix^24,27^. Quantifying the matrix contrast or quality for a species therefore requires comprehensive knowledge about the ecology and characteristics of the species as well the habitat-matrix landscape under study. This becomes even more challenging when entire species communities are considered. Nevertheless, it is important to move away from the less realistic binary view (habitat = hospitable, matrix = inhospitable), but rather develop gradual approaches^4^.

Closely linked to the question of prevailing isolating mechanisms and matrix contrast is the challenge of defining the species pool for species on habitat islands, which for true islands is the closest (or ecologically most similar) mainland, but much more difficult to define for habitat islands^26,28^. In some habitat islands, the species diversity could be predicted well if the distance to the largest and most species-rich habitat island in its vicinity was taken into account^26^. Thus, for some habitat island archipelagos (that is a spatial mosaic of similar habitat islands in an area), comparatively larger habitat islands appear to serve as a kind of mainland equivalent for some species^29^. Findings of other studies suggest that the surrounding matrix habitats can also act as a source for many of the species on a habitat island, especially when generalist species are included; and that the colonisation of habitat islands depends on the age of the island, similar to real islands^27,30^. However, while fluctuations in sea level provide a method to estimate important age-related patterns on true islands^31,32^ the past of habitat islands is often very heterogeneous with a complex human legacy often being superimposed upon natural drivers.

Several approaches have been proposed to address all these challenges by applying other models than the ETIB^33^. These approaches include (i) developing new parameters for fragmented ecosystems (such as the habitat amount hypothesis^22^), (ii) defining the degree of insularity by new isolation parameters^26^ or (iii) by indices that are based on area rather than distance^34^ , (iv) including matrix properties or defining the matrix contrast^4,35^, (v) excluding species other than habitat island specialists (often referred to as core island species^27,36–38^), or (vi) categorizing the habitat island system under study more specifically using classification criteria^5,20,21,39^.

Edaphic islands represent a subcategory within the broad concept of habitat islands in which demarcation from the surrounding area is created by changes in soil properties^5^. These islands, often occurring on special soils or outcrops, offer great opportunities to test the applicability of the previously described approaches, as natural patchiness is ideal for diversity studies^40^ and they often host diverse floras with high numbers of locally endemic or habitat-specialized species^41^, similar to true islands^42^. In addition, while there are several examples of such natural habitat islands, most fragmented landscapes globally are now the result of anthropogenic impacts^25,43^. Natural edaphic islands could therefore represent another, special type of islands with similarities to true oceanic islands but also anthropogenically caused habitat remnants. As a result, they might require a combined approach considering both, characteristics of true islands and more fragmented habitats, to study their biodiversity. The principle derived from biogeographical research on true islands that larger, continuous and less isolated areas harbour more species and should therefore be preferred to smaller, more fragmented areas for nature conservation has often been criticised by applied conservation research^22,44^. Furthermore, the matrix might play an equally important role in natural habitat island archipelagos; thus, protection of the surrounding landscape should be considered in concert with edaphic habitat islands.

Quartz islands are natural edaphic islands that are mainly distributed in the arid zones of southern Africa^45^. These edaphic islands are patches covered by quartz gravel and show distinct boundaries with the surrounding zonal habitats^46^; thus, they comprise an archipelago-like mosaic within the surrounding landscape^47^. Several habitat specialists and numerous local endemics evolved on these unique edaphic islands. The edaphic features such as a wide range in soil pH, partly high salinity and very shallow soil depth, as well as thermo-specific parameters, have already been described as important drivers for the remarkable plant diversity, unique life forms and high endemism^46,48,49^. The insular character of these particular areas and their potential for biogeographic questions have been recognized in previous studies^50,51^. However, the influence on the spatial arrangement and island biogeographic characteristics of quartz islands has not yet been individually quantified, and quartz islands have been underrepresented in previous research on edaphic islands as well as special soils and habitat islands in general.

Therefore, in this study, we examine the effects of selected island shape parameters (island area), different isolation/insularity measures (distance- and area-based isolation parameters), landscape parameters (habitat diversity), and a matrix contrast parameter developed specifically for this island system on quartz island plant diversity. We test different parameters developed for true island archipelagos and habitat island systems. In addition to the richness of generalist species, we also consider the richness and percentage of quartz specialists. Since the differences between the islands and their surroundings are apparent, we assume that plant diversity on the quartz islands can be predicted very well by the different island biogeographical parameters or those specifically adapted for habitat islands. Due to their contrasting edaphic properties, the Knersvlakte quartz patches are island-like and we hypothesize them to align with the predictions of the ETIB. However, we expect this island effect to vary between quartz-specialist and generalist species, as well as with the strength of the matrix-island contrast. In particular, we predict that (i) the species-area relationship will be stronger for quartz specialist compared to generalist species richness; (ii) species richness will increase with increasing habitat diversity, with this relationship being the most important for quartz specialists; (iii) matrix contrast and area-based isolation parameters will better predict the species richness on quartz islands as these parameters address the potential species pool.

## Material and methods

### Study area

**Fig. 1:**
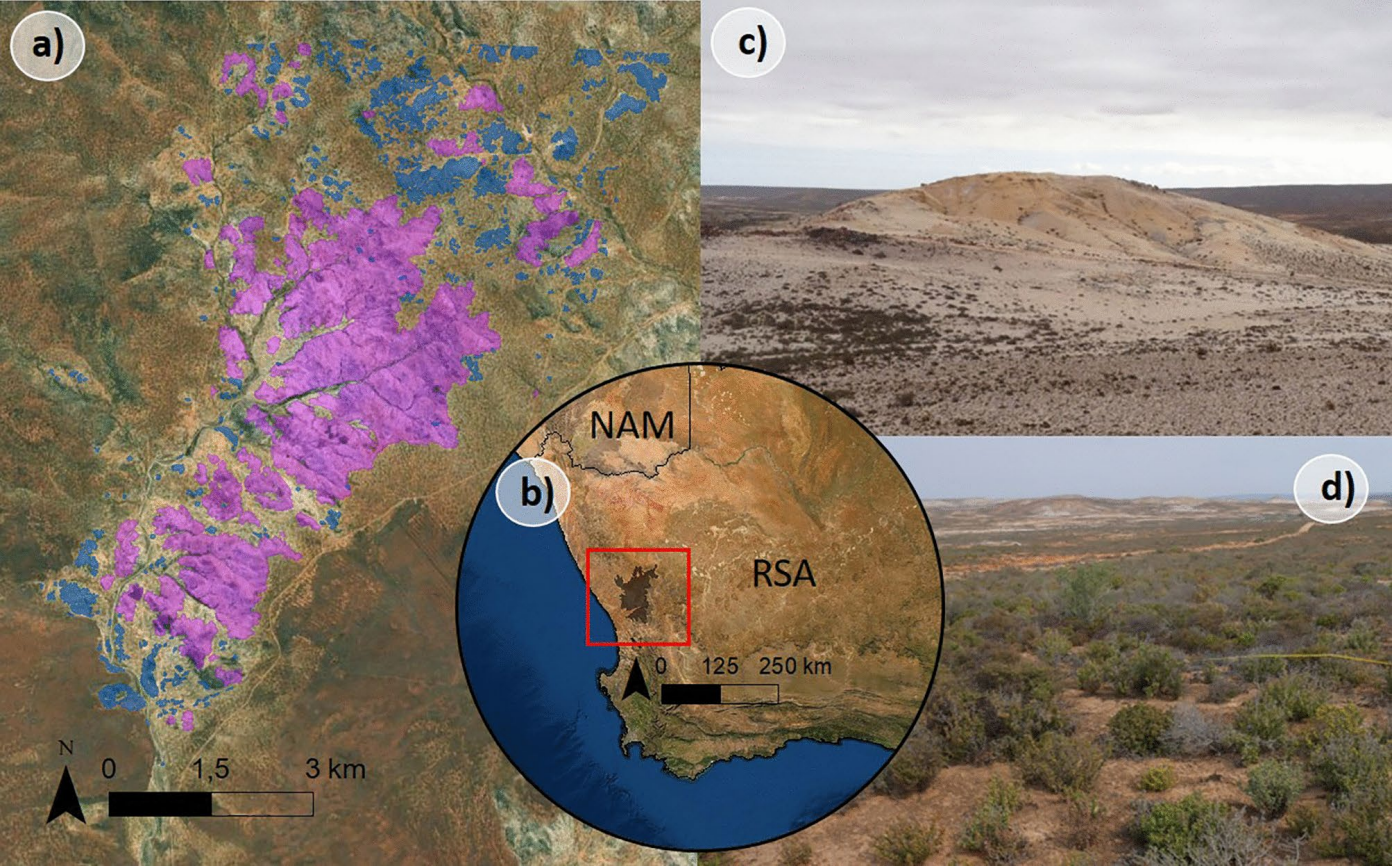
Map of the study area. a) The 47 sampled quartz islands (pink) and further quartz islands in the Moedverloren area (blue), b) the location of the Knersvlakte bioregion in the Western Cape of South Africa, c) quartz island covered with white quartz gravel, d) adjacent matrix habitats with higher and denser vegetation. (Photos: PE; Basemap sources: Esri, Digital Globe, GeoEye, Earthstar Geographics, CNES/Airbus DS, USDA, USGS, AeroGRID, IGN, and the GIS user community; Shapefiles: ^59^for the Knersvlakte shapefile; ^51^for the quartz island shapefiles).

The Knersvlakte, situated in the Western Cape of South Africa, is a bioregion that forms part of the Succulent Karoo biome. Spanning approximately 6300 km^2^, it features a gently rolling landscape^52^. The region falls within the winter rainfall area, experiencing rainfall exclusively during the wet season from May to September, and is characterized by high predictability and mild arid climatic conditions^53,54^. The vegetation in the area primarily comprises aridity-adapted plant species, with succulents being particularly prominent. The Succulent Karoo is recognized as a biodiversity hotspot, harboring approximately 5000 plant taxa, 40% of which are endemic^55^. Within the Knersvlakte bioregion, the landscape is characterized by quartz islands, also known as quartz fields or patches. These islands are of varying sizes and shapes and differ from the surrounding matrix habitats in terms of taxonomic composition, functional and phylo-diversity, as well as edaphic variables^30,41,47,49^. Quartz islands are formations derived from quartz veins in the underlying bedrock shaped by erosion processes over time^47,51,56,57^. They support a unique flora, consisting mainly of dwarf succulent species from the Aizoaceae, Crassulaceae, and Asteraceae families. This distinct flora contributes significantly to the overall diversity and endemism observed in the Knersvlakte^58^. Our study focused on the Moedverloren area, which is situated southwest of the Knersvlakte Nature Reserve and comprises a quartz island archipelago that is spatially isolated from other quartz archipelagos in the Knersvlakte and other sites in South Africa (Fig. 1).

### Sampling design: Island flora

Based on a pre-existing quartz island classification^51^, we randomly selected 47 quartz island polygons of different shapes and sizes with a range between 2551.4 m^2^ (min) and 8.3 km^2^ (max) in island area (Fig. 1a). Between August and October 2019, we sampled the complete floristic inventories with presence and absence recording of all selected islands (n = 47) by applying a systematic walk-through sampling approach (see^60^). Plant species were identified based on expert knowledge and the standard identification literature in this area^61–64^. For taxonomic nomenclature, we used the Red List of South African plants^65^ as a reference. We calculated the richness of generalist species (GR) for each island by subtracting the total number of species by the number of quartz specialist species. In addition, we calculated the richness of quartz specialists (QS) and the percentage of quartz specialists (QS %) per island. The classification of a species as a quartz specialist was based on whether it was identified as such in the relevant literature (e.g. “growing exclusively on quartz”, “found on white quartz pebbles”). In this regard, we mainly relied on the floras by^61,63^ and, for the Aizoaceae^64^. In the case of species not included in these floras or for which no information regarding the habitat preferences was provided, the individual original taxonomic publications were consulted. For all species that remained unclassified after this review of the literature, a decision was made based on the expertise and previous research findings of one of the authors (US; see also^41^ for detailed literature trait method description). It is important to note that a species’ quartz specialisation does not necessarily imply a restricted biogeographic distribution and, thus, classification as a local endemic. This is because quartz island archipelagos also exist in other parts of South Africa (see Supplementary figure S2 for details on endemic categories of the species in the study area).

#### Pre-analysis and predictor variable reduction

In a pre-analysis, we calculated several environmental parameters that were identified as important explanatory variables for species diversity or percentage endemism in studies on true islands (for example^15,66^) or edaphic islands^5,26,28,29,67^. All parameters were calculated for the 47 sampled islands, the calculation of the predictor variables considered also the remaining, unsampled quartz islands in the study area (Fig. 1). We grouped the parameters according to the processes or mechanisms into four groups: (i) *island shape parameters*, (ii) *habitat diversity indices*, (iii) *distance-based isolation parameters*, (iv) *area-based isolation parameters* and (v) a *matrix contrast index*. All predictor variables that were eliminated by the preliminary analysis are nevertheless described in detail in the Supplementary tables S1, S2 and Supplementary figure S1. The five predictors selected for the final analysis are described in detail in the following section. As we sampled the floristic inventory at the whole island-scale and not in equal-sized plots, we did not include the habitat amount^22^. However, some of the indices (e.g. *proximity index*) represent area-based isolation indices, and therefore can be considered as a similar measure to quantify the habitat amount in a local landscape. For the following analysis, we selected one predictor from each of the five categories as a proxy. This predictor variable reduction was based on the ecological significance of the predictor and on a correlation analysis between all predictor variables (see Supplementary table S2).

### Spatial predictor variables

The five final predictors selected for the following analysis were *island area* (*island shape parameters*), the *habitat diversity index* (*habitat diversity parameters*), the *distance to the nearest neighbouring island* (*distance-based isolation parameters*), the *target effect* (*area-based isolation parameters*) and the *matrix contrast index* (*matrix contrast parameters*).

#### Island shape parameter

*Island area* (measured in m^2^) was selected from the category *island shape parameters*, as it is one of the most important variables in the ETIB.

#### Habitat diversity parameter

The *habitat diversity index*^67^ was chosen as the parameter representing the habitat heterogeneity of each island. This index reflects the ratio of the total perimeter, which comprises the sum of the island perimeter and all boundaries between intra-island habitats, to the area of a specific island. This index captures potential edge or boundary effects both at the island-matrix interface and within the island itself^67^. To calculate the *habitat diversity index* of each island, we categorised the quartz island area into five main habitat types (ridges, slopes, plain, valley, drainages) with sub-habitats based on topographic parameters from a digital elevation model (slope, aspect, elevation) and the island matrix shapefiles from^51^ (see also Appendix for details and photo). We then calculated the total perimeter (TP) based on all the boundaries between habitats within the island and the boundary to the matrix around the island with the formula. We subsequently calculated the *habitat diversity index* by using the equation *TP*/(2 ∗ *sqrt*(π ∗ *A*))^67^ , where TP represents the total perimeter and A the island area.

#### Distance-based isolation parameter

We chose the distance to the nearest neighbour island, which we measured as the distance in metres from the boundary of the target island to the coast of the nearest island. Although the distance to the mainland is usually chosen in studies on true islands, there is no corresponding counterpart in habitat island archipelagos.

#### Area-based isolation parameter

Nevertheless, in order to test the effect of the largest and most species-rich island as a kind of mainland equivalent, we opted for the *target effect* in the category area-based isolation parameters. The *target effect* is a measure developed specifically for determining the degree of insularity of edaphic island systems and is calculated by the formula log(DMI/sqrt(A)) with DMI representing the distance to the main island (with the biggest island in the study area representing a kind of “mainland equivalent”) and A being the area of the target island. This parameter calculates the chances of colonization of the target island in relation to its size and distance from the species pool; high values of this index indicate a small island area or a large distance to the species pool and therefore smaller chances of colonization^29^. The *target effect* performed well in predicting the biodiversity on terrestrial habitat islands in former studies^26^.

#### Matrix contrast parameter

The final predictor was a *matrix contrast index* that we developed specifically for this study. This index quantifies how high the contrast to the matrix surrounding the island is in comparison to the respective island. We first calculated the normalized difference vegetation index (NDVI) for a multispectral WorldView-2 image (2 m resolution, taken during the August 2019 growing season, pre-processed by ImageBroker). We then classified the NDVI values into five units (Natural Breaks Jenks method). Using the pre-classification of quartz island polygons^51^, which integrates all quartz islands larger than 1000 m^2^ in area, we placed a 50 m buffer around each of the 47 sampled island polygons. This buffer traces the outline of the respective polygon with a distance of 50 m, which approximately aligns with the short distances in which most key plant groups mainly disperse^41,68^. For this buffer, we summed the NDVI values and then divided their sum by the area of the buffer to scale this value to the respective island size. High *matrix contrast* values imply that the island is surrounded by matrix habitats with denser vegetation, while low values indicate a rather open vicinity. This index quantifies potential dispersal barriers in the vicinity of island boundaries for plants, as the effects of island biogeographic (*area, isolation*) and landscape ecological (*matrix contrast*) parameters are strongly dependent on the dispersal characteristics of the species under study^69–71^. Most plants on the quartz islands, especially the key plant groups under study, tend to be short-distance dispersers (mostly ombrohydrochorous and autochorous species belonging mainly to the Asteraceae, Aizoaceae and Crassulaceae^41,68^) and require open, light-rich areas for germination^72^. Therefore, dense vegetation around the islands could act as an obstacle for successful germination because more generalist shrubs outcompete slow-growing dwarf succulents. This might increase the contrast effect of the matrix by reducing the permeability of the matrix, thus having an isolating effect on the dispersal of most species.

### Statistical analysis

The following analyses were performed in R version 4.2.1 (R Core Team 2022). We applied a power model (log (species richness)—log (island area); *lin_pow()* function in the sars package^73^) to calculate the species-area relationship (SAR) model for generalist species richness and quartz specialist richness. To test the applicability of the five included predictor variables (*island area, habitat diversity index, distance to the nearest neighbour island, target effect, matrix contrast*), we used single- and multi-predictor generalized linear models. The number of all generalist species (GR), the number of all quartz specialists (QS) and the percentage of habitat specialists (QS %) were included as untransformed variables as the respective response variable, and all five predictors were the explanatory variables. We checked for a normal distribution of the explanatory variables and used log10 transformations for non-normally distributed variables (variable + 0.01). We used a negative binomial distribution for the GLMs explaining generalist species richness and quartz specialist richness, as most models with a Poisson distribution showed overdispersion. We used a binomial distribution for GLMs to explain the percentage of quartz specialists. In the single-predictor models, we analysed for the linear as well the quadratic term. Before calculating the multi-predictor models, we standardized all predictor variables. Subsequently, we calculated the full multi-predictor models for generalist species richness, quartz specialist richness and quartz specialist percentage by including all five standardized explanatory variables either as linear or quadratic term in accordance with the results of the single-predictor models. We applied stepwise variable reduction to the full model according to the Akaike information criterion (AICc, dredge function in the MuMIn package^74^) and further excluded non-significant terms to obtain the final models. We controlled for potential collinearity and variance inflation by applying the vif function in the car package^75^. If the variance inflation factor indicated collinearity (VIF > 5) in the final model, we removed one of the collinear predictor variables. To estimate the explained variance of the model (Pseudo-R^2^), we divided the difference between the null deviance and the residual deviance by the null deviance of the respective model.

## Results

### Species-area relationship models of generalist richness and quartz specialist richness

**Fig. 2:**
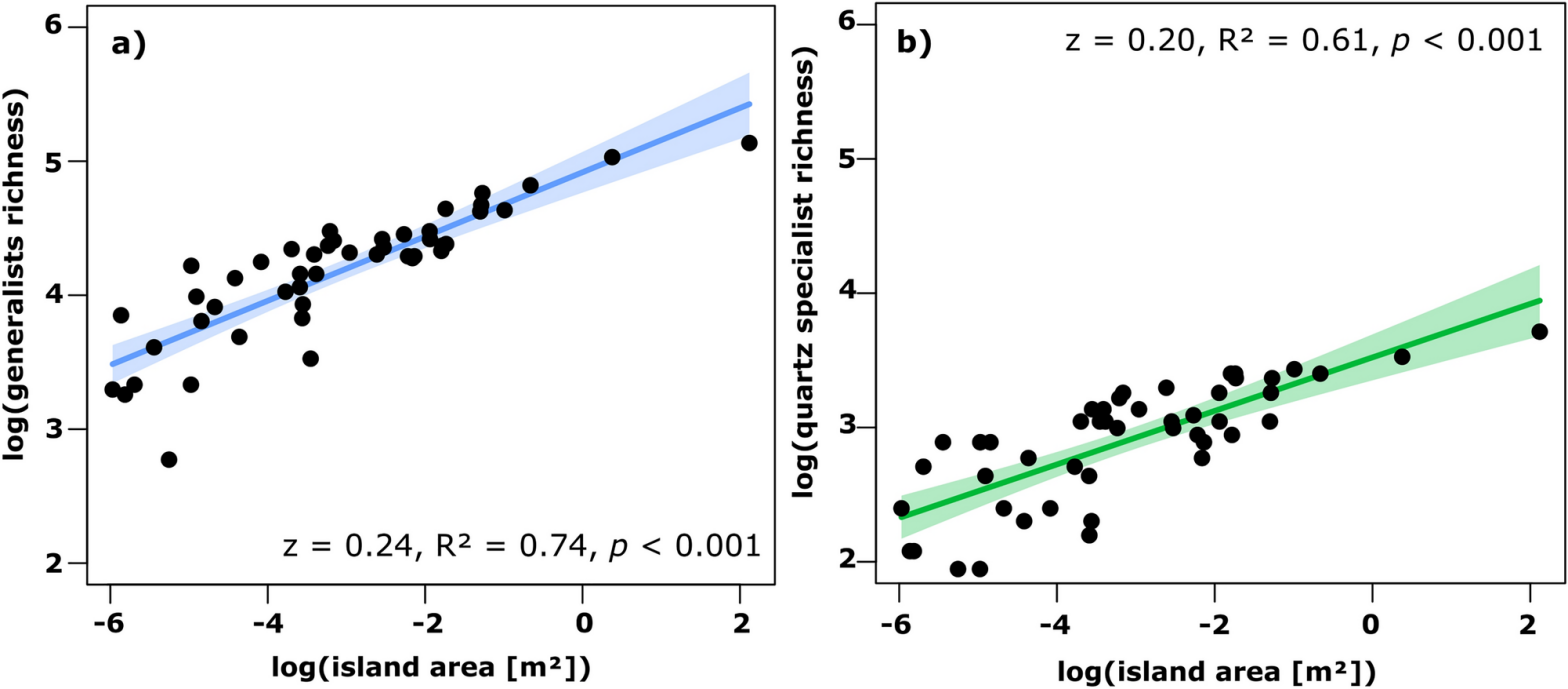
Power models of the relationship between island area and a) generalist richness and b) quartz specialist richness.

The power model of the generalist richness—area relationship showed a slightly higher z-value and higher explained variance than the quartz specialist richness—area model (Fig. 2). The power model of the generalist richness—area relationship explained a significant proportion of the variance (R^2^ = 0.74, F(1,45) = 127.5, *p* < 0.001) with a slope of 0.24 and a 95% confidence interval of [0.20, 0.28]. The power model of the quartz specialist richness—area relationship explained 61% of the variance (R^2^ = 0.61, F(1,45) = 69.3, *p* < 0.001) with the slope of 0.20 and a 95% confidence interval of [0.15, 0.25].

### Effects on generalist richness

**Fig. 3:**
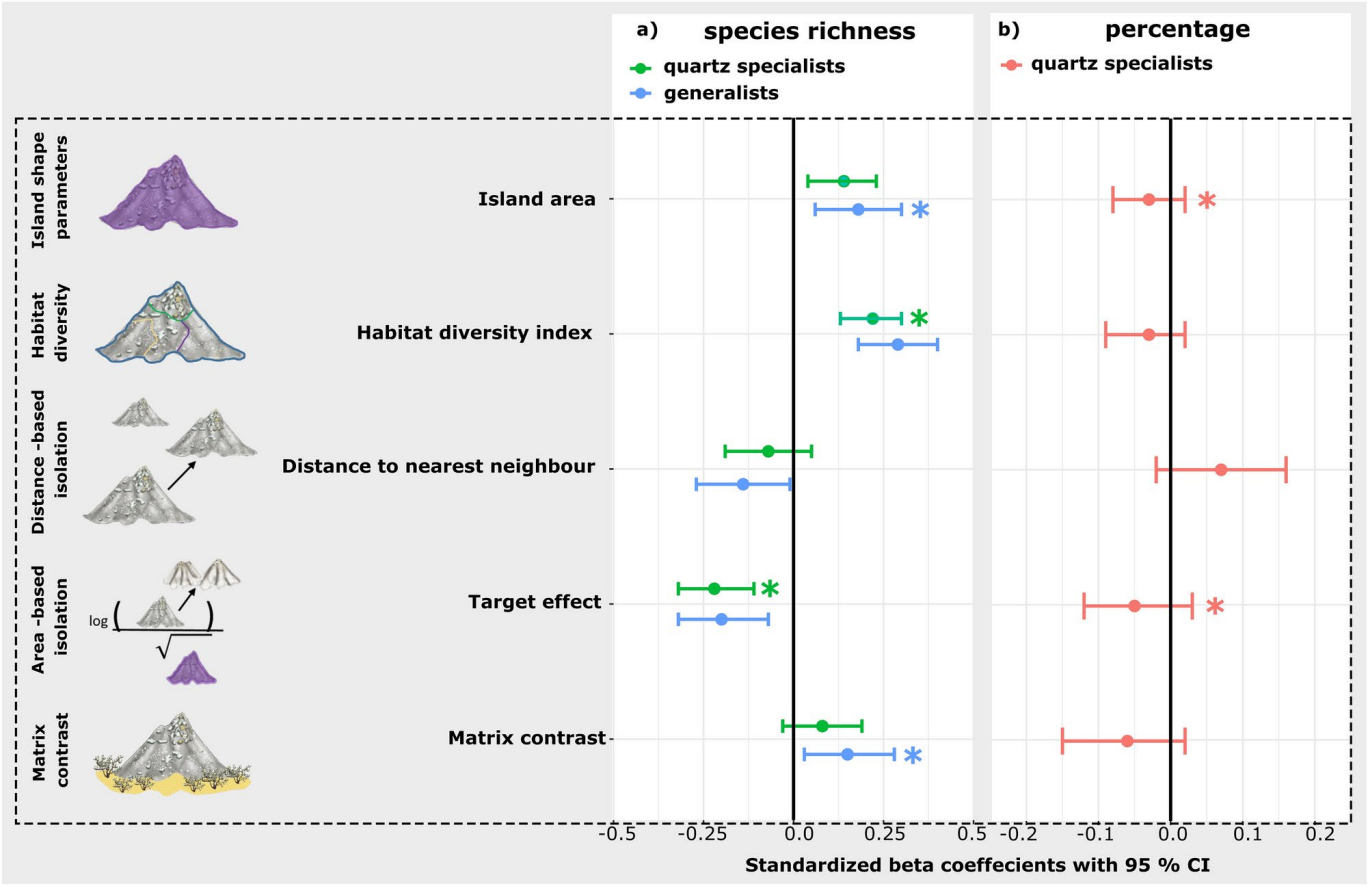
Standardized beta coefficient plots of the single-predictor models (untransformed but standardized explanatory variables) of a) generalist richness (blue), and quartz specialist richness (green) and b) the percentage of quartz specialists (orange). Stars indicate the significant variables that were included into the final model.

We found a total number of 434 vascular plant species across 47 sampled quartz islands. Generalist richness (n = 376) on the quartz islands significantly and linearly increased with an increase in the island area (log10 transformed, *p* < 0.001, Pseudo-R^2^ = 0.76) and an increase of the habitat diversity index (*p* < 0.001, Pseudo-R^2^ = 0.69). No significant relationship was found between generalist richness and the distance to the nearest neighbour island (log10 transformed). Generalist richness showed a significant hump-shaped relationship with increasing *target effect* (*p* < 0.001, Pseudo-R^2^ = 0.34) and increasing *matrix contrast* (*p* = 0.002, Pseudo-R^2^ = 0.25; details of single-predictor models in Supplementary table S3 and in Fig. 3). To calculate the final multi-predictor model, all five predictors were included into the full model with the *target effect* and *matrix contrast* as quadratic term (generalist richness ~ log10(*area*) + *habitat diversity index* + log10(*distance nearest neighbour*) + *target effect* + I(*target effect*^2) + *matrix contrast* + I(*matrix contrast*^2)). After the model selection (dredge function by AICc), the final model included the *island area* (log10) and the linear term of the *matrix contrast* as significant terms (*p* < 0.001, Pseudo-R^2^ = 0.81; details of the model selection in the Supplementary table S4 and figure S4). The variance inflation factor of the final model indicated no collinearity between the remaining predictors.

### Effects on quartz specialist richness

A total of 58 species were quartz habitat specialists. Quartz specialist richness on the quartz islands significantly increased with increasing values of the island area (log10 transformed, *p* < 0.001, Pseudo-R^2^ = 0.64) and the habitat diversity index (*p* < 0.001, Pseudo-R^2^ = 0.73), but slightly decreased with increasing distance to the nearest neighbour island (log10 transformed, *p* = 0.034, Pseudo-R^2^ = 0.08). Quartz specialist richness showed a significant, hump-shaped relationship with the *target effect* (*p* < 0.001, Pseudo-R^2^ = 0.50) and the *matrix contrast* (*p* = 0.0231, Pseudo-R^2^ = 0.13; details in Fig. 3 and Supplementary table S5). To calculate the final multi-predictor model, all five predictors were included into the full model with the *target effect* and *matrix contrast* as quadratic term (quartz specialist richness ~ log10(*area*) + *habitat diversity index* + log10(*distance nearest neighbour*) + *target effect* + I(*target effect*^2) + *matrix contrast* + I(*matrix contrast*^2)). After the model selection (dredge function by AICc), the final model included the habitat diversity index and the quadratic term of the *target effect* as significant terms (*p* < 0.001, Pseudo-R^2^ = 0.78; details of the model selection in the Supplementary table S6 and figure S5). The variance inflation factor of the final model indicated no collinearity between the remaining predictors.

### Effects on the percentage of quartz specialists

None of the single-predictor glms with the percentage of quartz specialists as response variable showed significant relationships (see Supplementary table S7). To calculate the final multi-predictor model, all five predictors were included into the full model as linear terms (quartz specialist percentage ~ log10(*area*) + *habitat diversity index* + log10(*distance nearest neighbour*) + *target effect* + *matrix contrast*). After the model selection (dredge function by AICc), the final model included the *island area*, the *habitat diversity index*, *matrix contrast* and *target effect*. However, as the variance inflation factor of this model indicated collinearity, we further reduced the final model by excluding the *habitat diversity index* and non-significant terms (*matrix contrast*). The final model for quartz species percentage contained the *island area* (*p* = 0.009) and the *target effect* (*p* = 0.02) as significant terms (Pseudo-R^2^ = 0.17; details of the model selection in the Supplementary table S8). The variance inflation factor of the final model indicated no collinearity between the remaining predictors.

To conclude, our results partly confirm our hypothesis that the diversity patterns on the quartz islands align with the predictions of the ETIB. As expected, species richness increased with increasing island area and habitat diversity for both, quartz specialists and generalists. As this relationship was similarly strong for both generalists and specialists, we can`t confirm our expectation of a stronger relationship for specialist richness, as predicted in (i). Regarding the other predictors, our results confirm our hypothesis that the relationship differs between generalists and quartz specialists. As predicted in (ii), habitat diversity had a stronger positive effect on the number of quartz specialists. As predicted in (iii), area-based isolation indices (*target effect*) and the *matrix contrast* performed better than distance-based isolation to capture the isolation of the habitat island archipelago under study. However, our results only partly align with the ETIB, as models including classical isolation parameters did not perform well.

## Discussion

This study is the first to explore the effects of island biogeographic and other spatial drivers on quartz island plants by introducing a previously underappreciated but highly suitable system for studying (habitat) island biogeography. In addition, we also developed an NDVI-based measure to define matrix contrast– a major research gap in the newly developing subdiscipline of habitat island biogeography^4^. Indeed, the insular nature of the quartz fields examined in this study has been frequently mentioned or assumed in previous research^47,49–51,76^ but has remained untested and unquantified until now. The main hypothesis that the ETIB is applicable to quartz islands has been partially confirmed by the results of our study. While the species-area relationship has been demonstrated to hold true for both generalist and quartz specialist species on quartz islands, the measurement of isolation remains a challenging endeavour.

### Island area is the dominating driver of generalist richness

Island area emerged as the strongest predictor of generalist richness in our analysis, which includes only “generalists” who may not depend exclusively on the unique edaphic conditions found on the quartz islands. The respective SAR power model underscores this relationship. The significance of the island area was expected, as the species-area relationship is one of the most well-established principles in (island) ecology^10,77,78^. Most habitat islands exhibit a positive correlation between species richness and island area, albeit somewhat weaker than in true islands; z-values are furthermore taxon-dependent^6,7,13,26^. Even in the model that includes only the number of quartz specialists, the quartz islands still display relatively high z-values comparable to the global average for habitat islands^13^. This suggests a potential isolation effect of the surrounding matrix on the quartz islands. However, the generally weaker relationship observed in other habitat islands, as indicated by lower z-values, can be attributed to lower levels of isolation in these systems than in true islands(e.g.^79,80^ and this study).

### Habitat diversity and edge effects drive richness of habitat specialists

Despite our initial hypothesis that habitat specialists would exhibit greater sensitivity to isolation and higher matrix contrasts, the species-area relationship in the power model focusing solely on quartz specialist richness was slightly weaker than those in the model considering generalist richness. When incorporating the habitat diversity index instead of island area as predictors, the model explaining quartz specialist richness displayed slightly stronger fits. This index captures potential edge or boundary effects both at the island-matrix interface and within the island itself^67^. It therefore reflects all habitat boundaries within an island, as well as those between the island and the surrounding habitat. This seems to reflect the heterogeneity apparent between the vegetation communities on the quartz islands, some of which are very sharply separated from one another^46^. The results of the models that included only the number of habitats per island as a proxy for habitat diversity were comparable to those of the models that included the habitat diversity index (see pre-analysis Supplementary figure S3). This indicates that habitat diversity has a significant impact on the flora of quartz islands. Most species within these communities exhibit strong habitat fidelity, which leads to distinct vegetation communities^46^. This results in high species turnover and clear boundaries between different vegetation communities on the quartz islands, ultimately driving high beta diversity^49^. Habitat diversity also emerged as an important predictor of plant diversity in other studies that have focused on habitat islands^81^. However, one challenge lies in identifying appropriate abiotic drivers underlying the habitat diversity of an edaphic island. In our study, we selected topographic heterogeneity, which is commonly employed and relatively easy to measure on islands^82^. While studies on true islands often focus on the percentage of endemics, we chose to focus on habitat specialists in this study. The percentage of habitat specialists is a diversity measure common in studies on edaphic islands^4,83^. Habitat specialists might play a more critical role compared to geographic endemics when examining the diversity within an archipelago of edaphic or habitat islands as they can better reflect the fine-scale nature of environmental changes at this scale^27,81,83,84^.

### The importance and challenges of including the matrix in edaphic island system studies

Addressing the role of the matrix is a challenge in landscape and habitat island ecology^4,24,69^; however, our results indicate that it might be worthwhile. Just as isolation is species-specific for island species^85^, quantifying matrix effects is highly taxon-dependent and becomes more complex when considering whole communities or even the entire island inventory, as in our study. This indicates that, for both groups, the highest richness is observed when the *matrix contrast* is of intermediate strength. This is contrary to the original assumption regarding habitat specialists, which suggested that the lowest *matrix contrast* would result in the greatest richness. However, a very low contrast in our index only reflects the NDVI, rather than the prevailing soil properties. To improve the *matrix contrast* quantification, soil properties should be incorporated in future studies together with vegetation data, for instance, via the NDVI. Interestingly, the matrix contrast index showed a significant hump-shaped, albeit relatively weak, correlation with the richness of generalist as well as quartz specialists. Considering matrix effects in habitat island studies might be a valuable addition, although defining a universal parameter that applies to all habitat island systems and taxa may pose challenges. Moreover, quantifying matrix contrast is not as straightforward as distance-based isolation measures and requires expertise in understanding the ecological traits of the target species or community. Future research on insularity in habitat island archipelagos would benefit from methodological advancements that move beyond a binary perspective of classifying islands and the matrix as suitable or unsuitable units and instead consider the heterogeneity of the surrounding landscape, recognizing matrix contrast as a species- and habitat-specific spectrum^4^.

### Promising predictors and limitations in edaphic island systems

In the studied quartz archipelago, landscape filters such as insularity parameters that are area- rather than distance-based appear to be better predictors, which agrees with the findings of studies on other habitat islands^26,28,29^ or fragmented ecosystems^23^. In other island systems where the distance between individual islands is small (compared to the very large distances in oceanic islands), it was more effective to measure isolation at the landscape level and consider it in combination with island size as a predictor^86^. Most area-related isolation indices included in the pre-analysis of this study showed a significant relationship with the diversity of the quartz island flora (see Supplementary figure S3). Some of the analysed indices are functionally similar to the habitat amount in the local landscape proposed in the habitat amount hypothesis^22^. Parameters quantifying the amount of suitable habitat in a landscape also emerged as good predictors in other patchy ecosystems^23^.

In our study, the *target effect* showed significant and good fits for generalist and quartz specialist richness, which brings us to the non-trivial question about the species pool (the *mainland* equivalence) for edaphic islands. The *target effect* uses the distance to the potential species source and has been reported as the best predictor in several studies on edaphic islands^26,28,29^. Thus, it could be inferred that despite its above-average species richness (n = 211), the main island, taken as the potential species source in the quartz archipelago, probably functions as a species pool, but not completely. Instead, the chance of colonization of a quartz island does not depend solely on the distance to the main island, but probably also on the close neighbourhood of other quartz islands. This suggests that evolutionary traits of the target species or community probably play a more critical role than considered so far and that one clearly defined mainland equivalent might not exist in a habitat island archipelago^22^. Furthermore, for some species, the surrounding matrix might phylogenetically resemble an additional source of species^30^.

### The importance of research on quartz islands—an understudied model system

The findings of our study provide evidence that quartz islands can be considered insular systems from a biogeographical perspective, although a different approach to the classical parameter of isolation is required. Island area and habitat diversity are reliable predictors of both generalist richness and quartz specialist richness. The results of the present study corroborate those of previous research, which indicated that habitat diversity is a particularly important predictor for habitat specialists on quartz islands^49^. Instead of relying solely on traditional distance-based isolation parameters, the concept of isolation or insularity in edaphic islands should incorporate area-based indices or measures that quantify the permeability of the matrix between islands, as in this study. Despite their unique edaphic and floristic characteristics and high conservation value, quartz islands have been underrepresented in (macro-)ecological studies focused on habitat islands and special soils. Including this type of edaphic island in future macroecological and island biogeographic research will undoubtedly provide new perspectives to habitat island biogeography. It is important to note that habitat islands are not entirely surrounded by a metaphorical "ocean," as implied by the title of this paper, but rather by a semi-permeable matrix with varying degrees of suitability, enabling certain species to pass through more easily than others do. Furthermore, the application of concepts from island biogeography or fragmented landscapes to edaphic islands raises questions that extend beyond the realm of basic science and align with the pursuit of answering fundamental questions in island biogeography research^87^.

### Implications for the conservation of the quartz island flora

Conservation planning for habitat islands and anthropogenically fragmented ecosystems often relies on findings from studies conducted on true islands, although direct transferability to terrestrial islands is not always straightforward, as demonstrated in the present study. Habitat diversity was found to be the most important predictor of high species richness on quartz islands in this study. This suggests that multiple small but heterogeneous islands can potentially harbour comparable numbers of species to a single large island, especially when these small islands are in close proximity, which is also a finding of other studies on habitat islands or outcrops^88^. The special role of the matrix and the minor importance of geographical isolation in quartz island archipelagos suggest that the entire quartz island archipelagos with their surrounding landscape should ideally be placed under protection. However, it is difficult to formulate a generalised strategy for all edaphic habitat islands because many other types of edaphic islands have not yet been studied from an island biogeographical perspective. However, the results of this study are consistent with other studies criticising the application of the ETIB on habitat islands. This indicates that a one-to-one comparison with oceanic islands and conclusions for nature conservation derived from this are not accurate and should therefore be treated with caution. An alternative interpretation of natural edaphic habitat islands, such as the quartz islands, is that they represent a distinct intermediate category of island. This category might be better understood by combining approaches from island biogeography and habitat island research. While most of the quartz islands in the Knersvlakte region studied here benefit from existing protection measures, other sites in the Succulent Karoo may face direct threats and potential irreversible damage owing to changes in land use and mining activities that target ores and minerals^89,90^. This is concerning considering the role of these edaphic islands as hotspots of unique taxonomic and functional plant diversity within the surrounding landscape.

## Supplementary Information

Supplementary Information 1.

Supplementary Information 2.

## Data Availability

The original dataset for this study is publicly available at the dryad repository at 10.5061/dryad.b2rbnzsnw.

## References

[1] MacArthur, R. H. & Wilson, E. O. *The Theory of Island Biogeography*. vol. 1 (JSTOR, 1967).

[2] Preston, F. W. The canonical distribution of commonness and rarity: Part I. *Ecology* **43**, 185–215 (1962).

[3] Laurance, W. F. Theory meets reality: how habitat fragmentation research has transcended island biogeographic theory. *Biological conservation* **141**, 1731–1744 (2008).

[4] Matthews, T. J. On The Biogeography of Habitat Islands: The Importance of Matrix Effects, Noncore Species, and Source-Sink Dynamics. *The Quarterly Review of Biology* **96**, 73–104 (2021).

[5] Itescu, Y. Are island-like systems biologically similar to islands?. *A review of the evidence. Ecography* **42**, 1298–1314 (2019).

[6] Dembicz, I. et al. Isolation and patch size drive specialist plant species density within steppe islands: a case study of kurgans in southern Ukraine. *Biodiversity and Conservation* **25**, 2289–2307 (2016).

[7] Dembicz, I. *et al.* Steppe islands in a sea of fields: Where island biogeography meets the reality of a severely transformed landscape. *J Veg Sci* **32**, (2021).

[8] Henneron, L., Sarthou, C., de Massary, J. & Ponge, J. Habitat diversity associated to island size and environmental filtering control the species richness of rock-savanna plants in neotropical inselbergs. *Ecography* **42**, 1536–1547 (2019).

[9] Zhang, S., Zhang, Q., Yan, Y., Han, P. & Liu, Q. Island biogeography theory predicts plant species richness of remnant grassland patches in the agro-pastoral ecotone of northern China. *Basic and Applied Ecology* **54**, 14–22 (2021).

[10] Scheiner, S. M. Six types of species-area curves. *Global ecology and biogeography* **12**, 441–447 (2003).

[11] Matthews, T. J. et al. Island species–area relationships and species accumulation curves are not equivalent: an analysis of habitat island datasets. *Global Ecology and Biogeography* **25**, 607–618 (2016).

[12] Watling, J. I. & Donnelly, M. A. Fragments as islands: a synthesis of faunal responses to habitat patchiness. *Conservation Biology* **20**, 1016–1025 (2006). 16922218 10.1111/j.1523-1739.2006.00482.x

[13] Matthews, T. J., Guilhaumon, F., Triantis, K. A., Borregaard, M. K. & Whittaker, R. J. On the form of species–area relationships in habitat islands and true islands. *Global Ecology and Biogeography* **25**, 847–858 (2016).

[14] Hortal, J., Triantis, K. A., Meiri, S., Thébault, E. & Sfenthourakis, S. Island species richness increases with habitat diversity. *The American Naturalist* **174**, E205–E217 (2009). 19857159 10.1086/645085

[15] Weigelt, P. & Kreft, H. Quantifying island isolation–insights from global patterns of insular plant species richness. *Ecography* **36**, 417–429 (2013).

[16] Keppel, G., Gillespie, T. W., Ormerod, P. & Fricker, G. A. Habitat diversity predicts orchid diversity in the tropical south-west Pacific. *Journal of Biogeography* **43**, 2332–2342 (2016).

[17] Stein, A., Gerstner, K. & Kreft, H. Environmental heterogeneity as a universal driver of species richness across taxa, biomes and spatial scales. *Ecology letters* **17**, 866–880 (2014). 24751205 10.1111/ele.12277

[18] Tamme, R., Hiiesalu, I., Laanisto, L., Szava-Kovats, R. & Pärtel, M. Environmental heterogeneity, species diversity and co-existence at different spatial scales. *Journal of Vegetation Science* **4**, 796-801 (2010).

[19] Ricketts, T. H. The Matrix Matters: Effective Isolation in Fragmented Landscapes. *The American Naturalist* **158**, 87–99 (2001). 18707317 10.1086/320863

[20] Watson, D. M. A conceptual framework for studying species composition in fragments, islands and other patchy ecosystems. *Journal of Biogeography* **29**, 823–834 (2002).

[21] Shepherd, U. L. & Brantley, S. L. Expanding on Watson’s framework for classifying patches: when is an island not an island?. *Journal of Biogeography* **32**, 951–960 (2005).

[22] Fahrig, L. Rethinking patch size and isolation effects: the habitat amount hypothesis. *J. Biogeogr.* **40**, 1649–1663 (2013).

[23] Watling, J. I. et al. Support for the habitat amount hypothesis from a global synthesis of species density studies. *Ecol Lett* **23**, 674–681 (2020). 32043741 10.1111/ele.13471

[24] Prevedello, J. A. & Vieira, M. V. Does the type of matrix matter? A quantitative review of the evidence. *Biodivers Conserv* **19**, 1205–1223 (2010).

[25] Ewers, R. M. & Didham, R. K. Confounding factors in the detection of species responses to habitat fragmentation. *Biol. Rev.* **81**, 117 (2005). 16318651 10.1017/S1464793105006949

[26] Mendez-Castro, F. E. et al. What defines insularity for plants in edaphic islands?. *Ecography* **44**, 1249–1258 (2021).

[27] Horsák, M. et al. The age of island-like habitats impacts habitat specialist species richness. *Ecology* **93**, 1106–1114 (2012). 22764496 10.1890/0012-9658-93.5.1106

[28] Conti, L. et al. Insularity promotes plant persistence strategies in edaphic island systems. *Global Ecology and Biogeography* **31**, 753–764 (2022).

[29] Ottaviani, G. et al. Sticking around: Plant persistence strategies on edaphic islands. *Diversity and Distributions* **28**, 1850–1862 (2022).

[30] Zhigila, D. A., Elliott, T. L., Schmiedel, U. & Muasya, A. M. Do phylogenetic community metrics reveal the South African quartz fields as terrestrial-habitat islands?. *Annals of Botany* **133**, 833–850 (2024). 38401154 10.1093/aob/mcae027 PMC11082514

[31] Steinbauer, M. J., Otto, R., Naranjo-Cigala, A., Beierkuhnlein, C. & Fernández-Palacios, J.-M. Increase of island endemism with altitude–speciation processes on oceanic islands. *Ecography* **35**, 23–32 (2012).

[32] Flantua, S. G. et al. Snapshot isolation and isolation history challenge the analogy between mountains and islands used to understand endemism. *Global Ecology and Biogeography* **29**, 1651–1673 (2020).

[33] Franklin, J. F. & Lindenmayer, D. B. Importance of matrix habitats in maintaining biological diversity. *Proceedings of the National Academy of Sciences* **106**, 349–350 (2009). 10.1073/pnas.0812016105 PMC2626705 19129497

[34] Gustafson, E. J. & Parker, G. R. Using an index of habitat patch proximity for landscape design. *Landscape and urban planning* **29**, 117–130 (1994).

[35] Wiser, S. K. & Buxton, R. P. Context matters: matrix vegetation influences native and exotic species composition on habitat islands. *Ecology* **89**, 380–391 (2008). 18409428 10.1890/07-0196.1

[36] Cook, W. M., Lane, K. T., Foster, B. L. & Holt, R. D. Island theory, matrix effects and species richness patterns in habitat fragments. *Ecol Letters* **5**, 619–623 (2002).

[37] Cook, W. M., Anderson, R. M. & Schweiger, E. W. Is the matrix really inhospitable? Vole runway distribution in an experimentally fragmented landscape. *Oikos* **104**, 5–14 (2004).

[38] Matthews, T. J., Cottee-Jones, H. E. & Whittaker, R. J. Habitat fragmentation and the species–area relationship: a focus on total species richness obscures the impact of habitat loss on habitat specialists. *Diversity and Distributions* **20**, 1136–1146 (2014).

[39] Watson, D. M. Continental islands. in *Encyclopedia of islands* (eds. Gillespie, R. & Clague, D. A.) 180–187 (Univ of California Press, 2009).

[40] Gastauer, M. et al. Landscape heterogeneity and habitat amount drive plant diversity in Amazonian canga ecosystems. *Landscape Ecology* **36**, 393–406 (2021).

[41] Eibes, P. M. et al. Testing the concept of edaphism for the quartz island flora of the Knersvlakte, South Africa. *South African Journal of Botany* **151**, 555–564 (2022).

[42] Stuessy, T. F. et al. Anagenetic evolution in island plants. *Journal of Biogeography* **33**, 1259–1265 (2006).

[43] Fahrig, L. Effects of Habitat Fragmentation on Biodiversity. *Annu. Rev. Ecol. Evol. Syst.* **34**, 487–515 (2003).

[44] Fahrig, L. Why do several small patches hold more species than few large patches?. *Global Ecol Biogeogr* **29**, 615–628 (2020).

[45] Schmiedel, U. The Quartz Fields of Southern Africa-flora, phytogeography, vegetation, and habitat ecology. (Universität zu Köln, 2002).

[46] Schmiedel, U., Kühne, N., Twerski, A. & Oldeland, J. Small-scale soil patterns drive sharp boundaries between succulent “dwarf” biomes (or habitats) in the arid Succulent Karoo, South Africa. *South African Journal of Botany* **101**, 129–138 (2015).

[47] Schmiedel, U. & Jürgens, N. Community structure on unusual habitat islands: quartz-fields in the Succulent Karoo. *South Africa.* **142**, 57–69 (1999).

[48] Schmiedel, U. & Jürgens, N. Habitat ecology of southern African quartz fields: studies on the thermal properties near the ground. *Plant Ecology* **170**, 153–166 (2004).

[49] Eibes, P. M. *et al.* Partitioned beta diversity patterns of plants across sharp and distinct boundaries of quartz habitat islands. *J Veg Sci* **32**, (2021).

[50] Musker, S. D., Ellis, A. G., Schlebusch, S. A. & Verboom, G. A. Niche specificity influences gene flow across fine-scale habitat mosaics in Succulent Karoo plants. *Mol Ecol* **30**, 175–192 (2021). 33152114 10.1111/mec.15721

[51] Oldeland, J., Eibes, P. M., Irl, S. D. H. & Schmiedel, U. Do image resolution and classifier choice impact island biogeographical parameters of terrestrial islands? *Transactions in GIS* (2022).

[52] Hoffman, M. T., Skowno, A., Bell, W. & Mashele, S. Long-term changes in land use, land cover and vegetation in the Karoo drylands of South Africa: Implications for degradation monitoring. *African Journal of Range & Forage Science* **35**, 209–221 (2018).

[53] Mucina, L. *et al.* Succulent Karoo Biome. in *The Vegetation of South Africa, Lesotho and Swaziland* (eds. Mucina, L. & Rutherford, M. C.) 221–299 (South African National Biodiversity Institute, 2006).

[54] Desmet, P. G. Namaqualand—a brief overview of the physical and floristic environment. *Journal of Arid environments* **70**, 570–587 (2007).

[55] Hilton-Taylor, C. Patterns and characteristics of the flora of the Succulent Karoo Biome, southern Africa. in *The biodiversity of African plants* 58–72 (Springer, 1996).

[56] Watkeys, M. K. Soils of the arid south-western zone of Africa. in *The Karoo: Ecological patterns and processes* (eds. Dean, W. R. J. & Milton, S.) 17–26 (Cambridge University Press, Cambridge, 1999).

[57] Curtis, O. E., Stirton, C. H. & Muasya, A. M. A conservation and floristic assessment of poorly known species rich quartz–silcrete outcrops within Rûens Shale Renosterveld (Overberg, Western Cape), with taxonomic descriptions of five new species. *South African Journal of Botany* **87**, 99–111 (2013).

[58] Hilton-Taylor, C. Western Cape Domain (Succulent Karoo). Republic of South Africa and Namibia. in *Centres of plant diversity. A guide and strategy for their conservation* vol. 1 204–217 (Oxford: Oxford University Press, Oxford, 1994).

[59] VEGMAP: The Vegetation Map of South Africa, Lesotho and Swaziland. (2006).

[60] Vetaas, O. R., Vikane, J. H., Saure, H. I. & Vandvik, V. North Atlantic Islands with native and alien trees: are there differences in diversity and species-area relationships?. *Journal of vegetation science* **25**, 213–225 (2014).

[61] Snijman, D. A. *Plants of the Greater Cape Floristic Region Volume 2: The Extra Cape Flora.* (South African National Biodiversity Institute, Pretoria, 2013).

[62] Fish, L., Mashau, A. C., Moeaha, M. J. & Nembudani, M. T. *Identification Guide to Southern African Grasses: An Identification Manual with Keys, Descriptions and Distributions.* (South African National Biodiversity Institute, 2015).

[63] Roux, A. *Wild Flowers of Namaqualand: A Botanical Society Guide*. (Penguin Random House South Africa, 2015).

[64] Hartmann, H. *Aizoaceae A-Z: Illustrated Handbook of Succulent Plants* (Springer, 2017).

[65] Raimondo, D. *et al. Red List of South African Plants 2009.* (South African National Biodiversity Institute, 2009).

[66] Schrader, J., Moeljono, S., Keppel, G. & Kreft, H. Plants on small islands revisited: The effects of spatial scale and habitat quality on the species–area relationship. *Ecography* **42**, 1405–1414 (2019).

[67] Patton, D. R. A diversity index for quantifying habitat" edge". *Wildlife Society Bulletin* **1973–2006**(3), 171–173 (1975).

[68] Parolin, P. Ombrohydrochory: Rain-operated seed dispersal in plants–With special regard to jet-action dispersal in Aizoaceae. *Flora-Morphology, Distribution, Functional Ecology of Plants* **201**, 511–518 (2006).

[69] Tscharntke, T. et al. Landscape moderation of biodiversity patterns and processes - eight hypotheses. *Biological Reviews* **87**, 661–685 (2012). 22272640 10.1111/j.1469-185X.2011.00216.x

[70] Cote, J. et al. Evolution of dispersal strategies and dispersal syndromes in fragmented landscapes. *Ecography* **40**, 56–73 (2017).

[71] Walentowitz, A., Troiano, C., Christiansen, J. B., Steinbauer, M. J. & Barfod, A. S. Plant dispersal characteristics shape the relationship of diversity with area and isolation. *Journal of Biogeography* **49**, 1599–1608 (2022).

[72] Schmiedel, U., Siemen, S.-E., Dludlu, M. N. & Oldeland, J. Germination success of habitat specialists from the Succulent Karoo and Renosterveld on different soil types. *South African Journal of Botany* **137**, 320–330 (2021).

[73] Matthews, T. J., Triantis, K. A., Whittaker, R. J. & Guilhaumon, F. sars: an R package for fitting, evaluating and comparing species–area relationship models. *Ecography* **42**, 1446–1455 (2019).

[74] Barton, K. MuMIn: Multi-model inference. R package version 1.7. 2. https://CRAN.R-project.org/package=MuMIn (2012).

[75] Fox, J. *et al.* car: Companion to Applied Regression. R package version 3.0–2. https://CRAN.R-project.org/package=car [accessed 17 March 2020] (2019).

[76] Schmidt, S. A., Carstens, F., Rau, A.-L. & Schmiedel, U. Diversity on a small scale – phylogeography of the locally endemic dwarf succulent genus Oophytum N.E.Br. (Aizoaceae) in the Knersvlakte of South Africa. *Annals of Botany* (in press). 10.1093/aob/mcae207 PMC11904907 39656776

[77] Kalmar, A. & Currie, D. J. A global model of island biogeography. *Global Ecology and Biogeography* **15**, 72–81 (2006).

[78] Whittaker, R. J., Fernández-Palacios, J. M., Matthews, T. J., Borregaard, M. K. & Triantis, K. A. Island biogeography: taking the long view of nature’s laboratories. *Science* **357**, eaam8326 (2017). 10.1126/science.aam8326 28860356

[79] Triantis, K. A., Guilhaumon, F. & Whittaker, R. J. The island species-area relationship: biology and statistics: The island species-area relationship. *Journal of Biogeography* **39**, 215–231 (2012).

[80] Fattorini, S., Borges, P. A., Dapporto, L. & Strona, G. What can the parameters of the species–area relationship (SAR) tell us? Insights from Mediterranean islands. *Journal of Biogeography* **44**, 1018–1028 (2017).

[81] Yan, Y. et al. Habitat heterogeneity determines species richness on small habitat islands in a fragmented landscape. *Journal of Biogeography* **50**, 976–986 (2023).

[82] Stein, A. & Kreft, H. Terminology and quantification of environmental heterogeneity in species-richness research. *Biological Reviews* **90**, 815–836 (2015). 25099766 10.1111/brv.12135

[83] Matthews, T. J., Steinbauer, M. J., Tzirkalli, E., Triantis, K. A. & Whittaker, R. J. Thresholds and the species-area relationship: a synthetic analysis of habitat island datasets. *J. Biogeogr.* **41**, 1018–1028 (2014).

[84] Deák, B. et al. Landscape and habitat filters jointly drive richness and abundance of specialist plants in terrestrial habitat islands. *Landscape Ecol* **33**, 1117–1132 (2018).

[85] Steinbauer, M. J. et al. Plant invasion and speciation along elevational gradients on the oceanic island La Palma. *Canary Islands. Ecol Evol* **7**, 771–779 (2017). 28116071 10.1002/ece3.2640 PMC5243188

[86] Diver, K. C. Not as the crow flies: assessing effective isolation for island biogeographical analysis. *Journal of Biogeography* **35**, 1040–1048 (2008).

[87] Patiño, J. et al. A roadmap for island biology: 50 fundamental questions after 50 years of The Theory of Island Biogeography. *Journal of Biogeography* **44**, 963–983 (2017).

[88] Wintle, B. A. et al. Global synthesis of conservation studies reveals the importance of small habitat patches for biodiversity. *Proc Natl Acad Sci USA* **116**, 909–914 (2019). 30530660 10.1073/pnas.1813051115 PMC6338828

[89] Rouget, M., Richardson, D. M., Cowling, R. M., Lloyd, J. W. & Lombard, A. T. Current patterns of habitat transformation and future threats to biodiversity in terrestrial ecosystems of the Cape Floristic Region. *South Africa. Biological Conservation* **112**, 63–85 (2003).

[90] Brownlie, S. et al. Systematic conservation planning in the Cape Floristic Region and Succulent Karoo, South Africa: enabling sound spatial planning and improved environmental assessment. *Journal of Environmental Assessment Policy and Management* **7**, 201–228 (2005).

