# Supplementary material for: Specific island biogeographic and landscape features shape plant diversity and habitat specialism on edaphic quartz islands in an arid ocean
Source: Sci Rep. 2025 Mar 22;15:9982. doi: 10.1038/s41598-025-94562-5 (PMC11929794; doi:10.1038/s41598-025-94562-5)
Supplement: Supplementary file 2 — Supplementary Information 2. [file 41598_2025_94562_MOESM2_ESM.docx]

**Supplemental Information for:**

**Specific island biogeographic and landscape features shape plant diversity and habitat specialism on edaphic quartz islands in an arid ocean**

**Scientific Reports**

PM Eibes, U Schmiedel, J Oldeland, SDH Irl. Corresponding author and email: Pia Maria Eibes, eibes@geo.uni-frankfurt.de

**Table of Contents:**

| **Table S1: Overview Indices Pre-Analysis** | Page 2-3 |
| --- | --- |
| **Table S2: Correlation Matrix Pre-Analysis** | Page 4 |
| **Figure S1: Overview Indices Pre-Analysis** | Page 5 |
| **Figure S2: Venn diagram endemic species** | Page 6 |
| **Figure S3: Pre-analysis of all predictors** | Page 7 |
| **Figure S4: Scatterplots non-linear predictors GR** | Page 8 |
| **Figure S5: Scatterplots non-linear predictors QR** | Page 11 |
| **Table S3: Model output single-predictor glms** | Page 9 |
| **Table S4: Model output multi-predictor glms** | Page 10 |
| **Table S5: Model output single-predictor glms** | Page 12 |
| **Table S6: Model output multi-predictor glms** | Page 13 |
| **Table S7: Model output single-predictor glms** | Page 14 |
| **Table S8: Model output multi-predictor glms** | Page 15 |

Tab. S1: Overview of the 15 metrics studied as predictors of the richness (total richness, quartz specialist and endemic richness) and the percentage of endemics and quartz specialists of vascular plant species on 47 quartz islands. For a better overview, the parameters were classified into four groups representing the characteristic of the island itself (island parameters), the habitat diversity on the island (habitat diversity) and the insularity of an island (distance-based, area-based and matrix-contrast indices). All indices were calculated using the island classification of Oldeland et al., 2022 (WorldView2-imagery). Habitat richness was defined as topographic heterogeneity by building 9 different habitat classes on the quartz islands based on a DEM: valley, plain, open slope, upper slope, midslope drainage, upland drainage, high ridge, local ridge, midslope ridge). The five parameters for the final analysis are highlighted in bold.

| **Group of metric** | **Abbreviation** | **Index** | **Formula** | **Unit** | **References & Notes** |
| --- | --- | --- | --- | --- | --- |
| **Island shape parameters** | **A** | **Area** |  | m² |  |
|  | P | Perimeter |  | m |  |
|  | SHP | Shape Index | P/(2*sqrt(3.1415 * A)) |  | Schrader 2019, according to Patton 1975 just with P instead of TP |
| **Habitat diversity** | HABRICH | Habitat richness |  |  |  |
|  | HABSHAN | Shannon-Wiener of habitat richness |  |  |  |
|  | **DI** | **Habitat Diversity index** | TP/(2*sqrt(3.1415 * A)) |  | Patton 1975; with TP as total perimeter (including also the edges between habitats within the islands) |
| **Insularity (distance-based)** | **DNNI** | **Distance to nearest neighboring Island** |  | m | “coast” to “coast” distance |
|  | DNSI | Distance to the nearest similarly large island |  | m | “coast” to “coast” distance |
|  | DMI | Distance to the main island (assuming it is the largest) |  | m | “coast” to “coast” distance |
|  | DNII | Distance to nearest other sampled island |  | m | “coast” to “coast” distance |
| **Insularity**  **(area-based)** | ANNI | Area of the nearest neighboring island |  | m² |  |
|  | NI | Neighbour index (in km²) is similar to ANNI when D is very small | Sum(A/(D+1)^2 |  | Kalmar & Currie 2006 |
|  | PX | Proximity index with 4000 as buffer size | Sum of all n(A/z) |  | Gustafson & Parker 1994 |
|  | **TE** | **Target effect** | log(DMI/sqrt(A)) |  | Mendez-Castro et al. 2021 |
| **Insularity**  **(matrix contrast)** | **MC** | **Matrix contrast index** | MC was developed for this study:  To address the surrounding matrix, we developed a matrix contrast index (MC). To do this, we first calculated the normalized difference vegetation index (NDVI) for a multispectral WorldView-2 image (2 m resolution, taken during the August 2019 growing season, pre-processed by ImageBroker). We then classified the NDVI values into five units (Natural Breaks Jens method). Using the quartz island polygons classified by Oldeland et al. (2022), which integrates all quartz islands larger than 1000 m² in area, we placed a 50 m buffer around each of the 47 sampled island polygons (a buffer that traces the outline of the respective polygon with a distance of 50 m). For this buffer, we summed the NDVI values and then divided their sum by the area of the buffer to scale this value to the respective island size. | | |
|  |  |  |  |  |  |

Tab S2: Pearson-Correlation matrix of the 15 predictor variables (A = Island area, P = Island perimeter, SHP = Shape index, DNNI = Distance to nearest neighboured island, ANNI = Area of nearest neighboured island, NI = Neighbour Index, DI = Diversity Index, DNII_47= Distance to nearest sampled island, DNSI = Distance to nearest similarly large island, PX = Proximity Index, DMI = Distance to main island, HABRICH = number of habitats, HABSHAN = Shannon-Wiener-Index using habitats, MX= Matrix contrast index, TE= Target effect). The island parameters island area (A), island perimeter (P) and the shape index (SHP) showed high positive collinearity with each other as well as with the diversity index (DI), the neighbor index (NI) and in the case of island area also with habitat richness (HABRICH). The habitat heterogeneity indices (DI, HABRICH, HABSHAN) were also positively collinear with each other and with the neighbor index (NI). The distance to the main island (DMI) was positively collinear with the target effect (TE), and the distance to the nearest island (DNNI) showed high positive collinearity with the distance to the nearest sampled island (DNII).

|  | **A** | **P** | **SHP** | **DNNI** | **ANNI** | **NI** | **DI** | **DNII_47** | **DNSI** | **PX** | **DMI** | **HABRICH** | **HABSHAN** | **MC** | **TE** |
| --- | --- | --- | --- | --- | --- | --- | --- | --- | --- | --- | --- | --- | --- | --- | --- |
| **A** | 1 | 1 | 0,8 | -0,3 | -0,1 | 1 | 0,9 | -0,3 | -0,3 | 0,1 | -0,2 | 0,7 | 0,5 | 0,1 | -0,4 |
| **P** | 1 | 1 | 0,9 | -0,3 | -0,1 | 1 | 0,9 | -0,3 | -0,3 | 0,2 | -0,2 | 0,7 | 0,5 | 0,2 | -0,4 |
| **SHP** | 0,8 | 0,9 | 1 | -0,3 | -0,2 | 0,8 | 0,8 | -0,3 | -0,3 | 0,2 | -0,2 | 0,5 | 0,5 | 0,2 | -0,4 |
| **DNNI** | -0,3 | -0,3 | -0,3 | 1 | 0 | -0,3 | -0,3 | 0,7 | 0 | -0,2 | 0 | -0,3 | -0,3 | 0,1 | 0,1 |
| **ANNI** | -0,1 | -0,1 | -0,2 | 0 | 1 | 0 | 0 | 0,1 | -0,1 | 0,2 | -0,4 | -0,1 | 0,1 | -0,1 | -0,5 |
| **NI** | 1 | 1 | 0,8 | -0,3 | 0 | 1 | 0,9 | -0,3 | -0,3 | 0,2 | -0,2 | 0,6 | 0,5 | 0,1 | -0,4 |
| **DI** | 0,9 | 0,9 | 0,8 | -0,3 | 0 | 0,9 | 1 | -0,3 | -0,3 | 0,3 | -0,2 | 0,8 | 0,8 | 0,2 | -0,4 |
| **DNII_47** | -0,3 | -0,3 | -0,3 | 0,7 | 0,1 | -0,3 | -0,3 | 1 | 0,2 | -0,3 | 0 | -0,2 | -0,2 | 0,1 | 0,1 |
| **DNSI** | -0,3 | -0,3 | -0,3 | 0 | -0,1 | -0,3 | -0,3 | 0,2 | 1 | -0,2 | 0,2 | -0,3 | -0,3 | -0,2 | 0,2 |
| **PX** | 0,1 | 0,2 | 0,2 | -0,2 | 0,2 | 0,2 | 0,3 | -0,3 | -0,2 | 1 | -0,4 | 0,2 | 0,5 | 0 | -0,4 |
| **DMI** | -0,2 | -0,2 | -0,2 | 0 | -0,4 | -0,2 | -0,2 | 0 | 0,2 | -0,4 | 1 | -0,1 | -0,1 | -0,2 | 0,7 |
| **HABRICH** | 0,7 | 0,7 | 0,5 | -0,3 | -0,1 | 0,6 | 0,8 | -0,2 | -0,3 | 0,2 | -0,1 | 1 | 0,8 | 0,2 | -0,3 |
| **HABSHAN** | 0,5 | 0,5 | 0,5 | -0,3 | 0,1 | 0,5 | 0,8 | -0,2 | -0,3 | 0,5 | -0,1 | 0,8 | 1 | 0,3 | -0,3 |
| **MC** | 0,1 | 0,2 | 0,2 | 0,1 | -0,1 | 0,1 | 0,2 | 0,1 | -0,2 | 0 | -0,2 | 0,2 | 0,3 | 1 | 0,1 |
| **TE** | -0,4 | -0,4 | -0,4 | 0,1 | -0,5 | -0,4 | -0,4 | 0,1 | 0,2 | -0,4 | 0,7 | -0,3 | -0,3 | 0,1 | 1 |


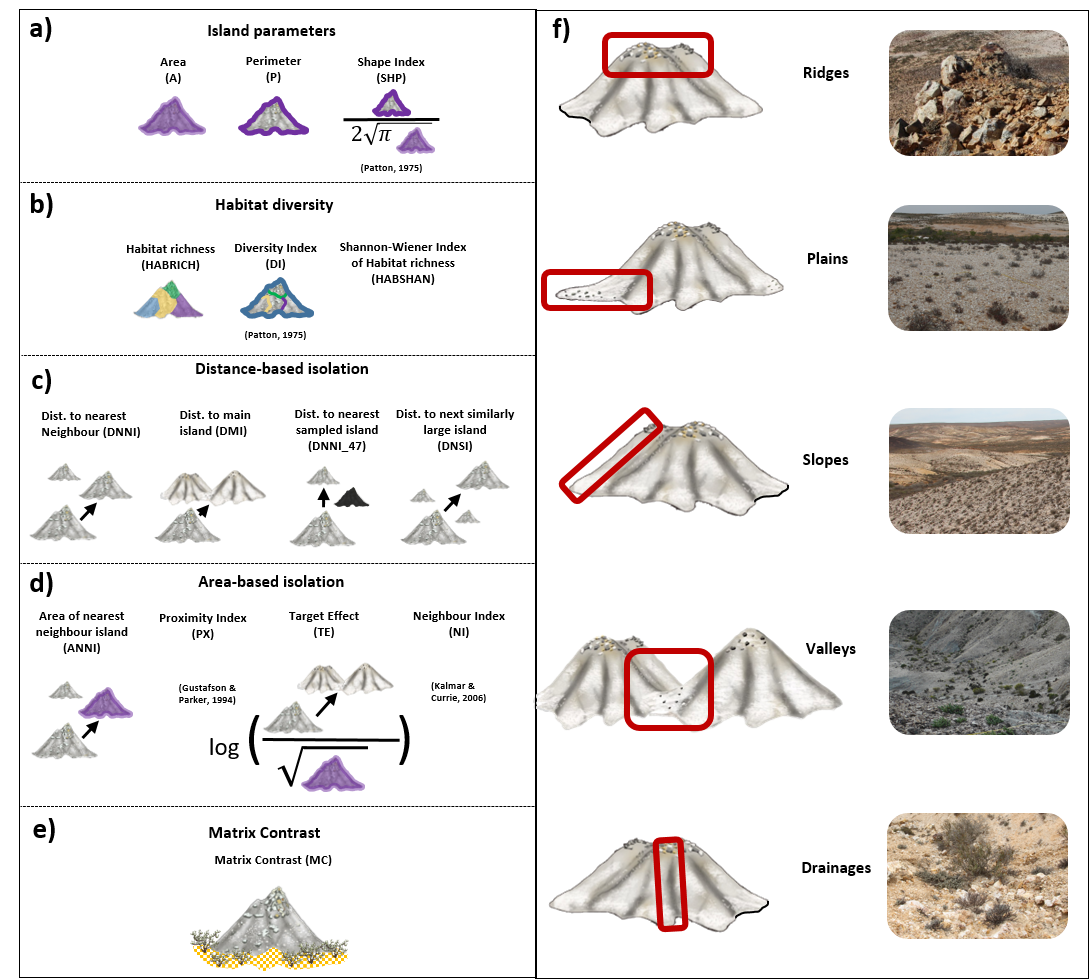


Fig. S1: Overview of all explanatory variables included in the pre-analysis and grouped into five different categories. A) Island shape parameters represent spatial features of the individual island such as island area; b) Habitat diversity calculates the topographic heterogeneity of each island; c) Distance-based isolation measures measure the spatial isolation of each island, while d) area-based isolation measures include other features such as area besides isolation; e) the matrix contrast was developed specifically for the island system under study; f) examples of the five main habitats on quartz islands according to topographic characteristics (illustrations and pictures by Eibes 2024 modified for this figure).


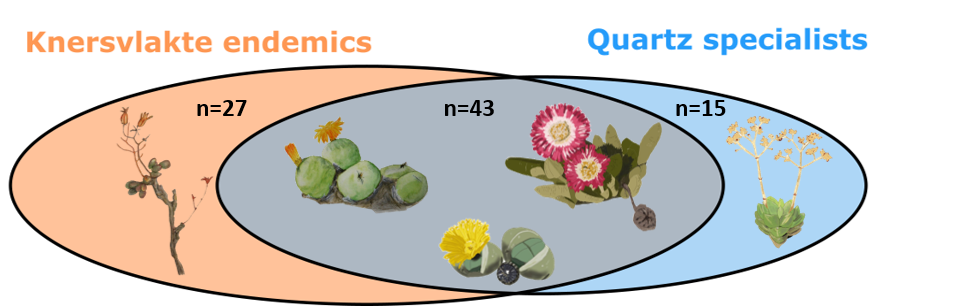


Fig. S2: Venn diagram showing the overlap between species endemic to the Knersvlakte (orange, n=70, e.g. *Tylecodon tenuis*) and quartz specialized species (blue, n=58, e.g. *Crassula deceptor*) with 43 species belonging to both categories (grey, e.g. *Conophytum calculus subsp. calculus*, *Argyroderma crateriforme*, *Cephalophyllum spissum*, Illustrations by Pia Eibes).


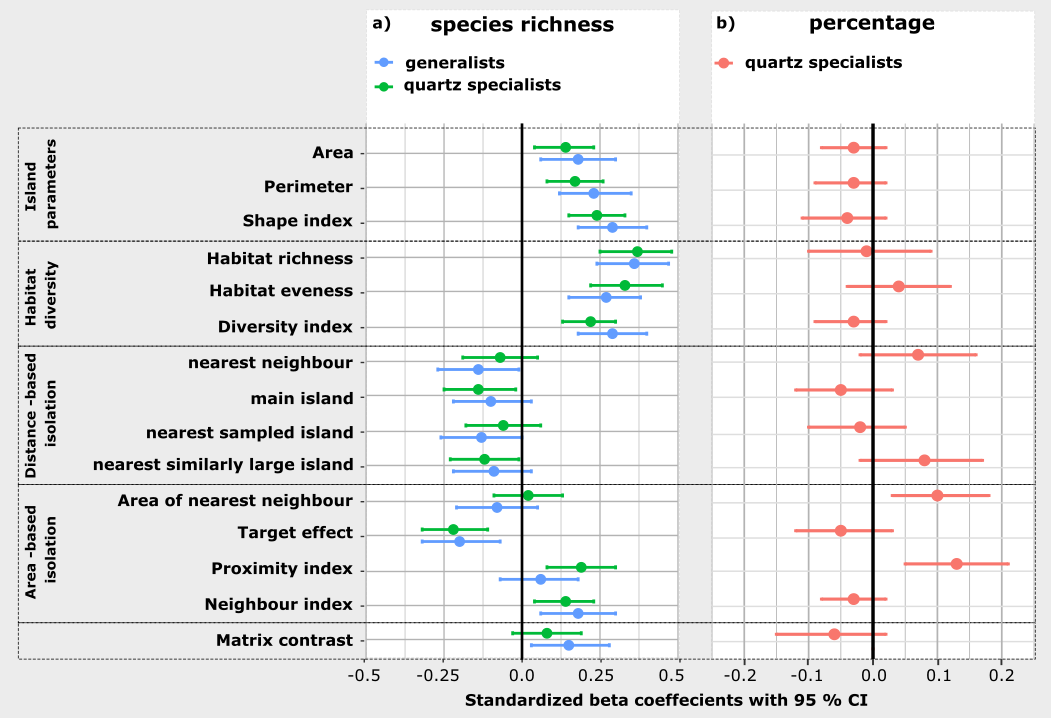


Fig. S3: Standardized beta coefficient plots of the single-predictor models (untransformed but standardized explanatory variables) of a) generalists richness (blue) and quartz specialist richness (green) and b) the percentage of quartz specialists (red). The figure shows the results of the pre-analysis used for variable selection for the final analysis. As most variables within the same category showed high similarity, we decided to select one predictor out of each category to be incorporated into the final analysis.


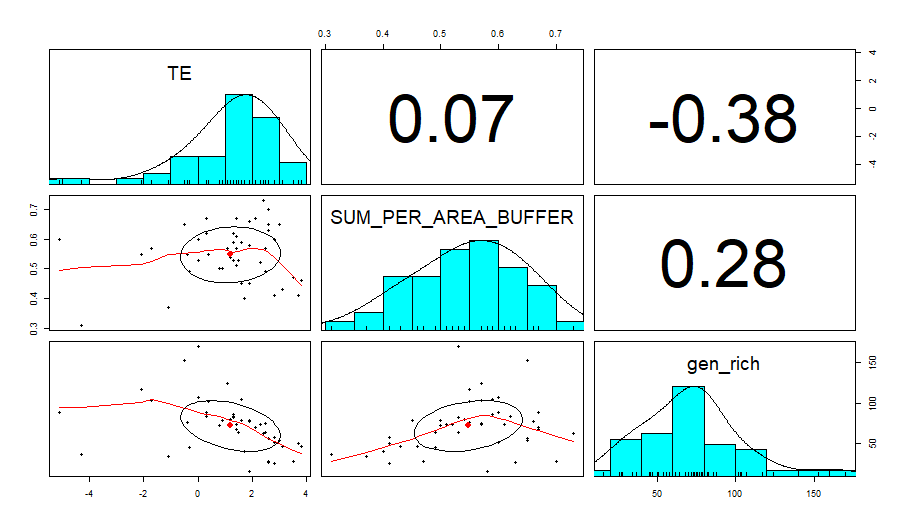


Fig. S4: Pairs-panel figure with the histograms (blue), scatterplots with loess-curves (scatterplots with red line) and the correlation coefficient of the generalist richness (gen_rich), the target effect (TE) and the matrix contrast (SUM_PER_AREA_BUFFER). The hump-shaped scatterplot between the generalist richness and the matrix contrast indicated non-linear relationship between these variables.

Tab S3: Model outputs for single-predictor generalized linear models (glms) for the generalist richness (number of all vascular plant species minus the quartz specialist species) and the 5 final predictor variables. We used negative binomial distribution for all models, as most models with Poisson distribution showed overdispersion. To estimate the explained variance of the model (Pseudo-R²), we divided the difference between the null deviance and the residual deviance by the null deviance of the respective model.

|  |  | **Predictor** | **Transformation** | **Index** | **n** | **AIC** | **p-value** | **sign. Level** | **Pseudo-R** |
| --- | --- | --- | --- | --- | --- | --- | --- | --- | --- |
| **Island parameters** | | |  |  |  |  |  |  |  |
| Generalists | ~ | A | log10 | Island area | 47 | 389.26 | <0.001 | *** | 0.76 |
| **Habitat diversity** | | |  |  |  |  |  |  |  |
| Generalists | ~ | DI |  | Habitat diversity index | 47 | 402.08 | <0.001 | *** | 0.69 |
| **Isolation metrics (distance-based)** | | | |  |  |  |  |  |  |
| Generalists | ~ | DNNI | log10 | Distance to nearest neighbour island | 47 | 454.77 | 0.06 | ns | 0.065 |
| **Isolation metrics (area-based)** | | | |  |  |  |  |  |  |
| Generalists | ~ | TE |  | Target Effect | 47 | 440.2 | <0.001 | *** | 0.34 |
| **Matrix contrast** | |  |  |  |  |  |  |  |  |
| Generalists | ~ | MC |  | Matrix Contrast | 47 | 446.35 | 0.002 | ** | 0.25 |

Tab S4: Model outputs by the dredge function for the first 20 multi-predictor generalized linear models (glms) for the generalist species richness (number of all vascular plant species minus the quartz specialist species) and the 5 final predictor variables. We used negative binomial distribution for all models, as most models with Poisson distribution showed overdispersion. The first row shows the final model (grey).

| **Model No** | **(Intercept)** | **A** | **DI** | **DNNI** | **MC** | **I(MC^2)** | **TE** | **I(TE^2)** | **df** | **logLik** | **AICc** | **delta** | **weight** |
| --- | --- | --- | --- | --- | --- | --- | --- | --- | --- | --- | --- | --- | --- |
| 10 | 4,20 | 0,37 |  |  | 0,09 |  |  |  | 4,00 | -186,77 | 382,50 | 0,00 | 0.28 |
| 26 | 4,19 | 0,38 |  |  | 0,10 | 0,01 |  |  | 5,00 | -186,70 | 384,86 | 2,36 | 0.09 |
| 74 | 4,19 | 0,37 |  |  | 0,10 |  |  | 0,00 | 5,00 | -186,74 | 384,95 | 2,45 | 0.08 |
| 42 | 4,20 | 0,37 |  |  | 0,09 |  | 0,00 |  | 5,00 | -186,76 | 384,99 | 2,49 | 0.08 |
| 14 | 4,20 | 0,37 |  | 0,00 | 0,09 |  |  |  | 5,00 | -186,77 | 385,00 | 2,49 | 0.08 |
| 12 | 4,20 | 0,36 | 0,01 |  | 0,09 |  |  |  | 5,00 | -186,77 | 385,00 | 2,50 | 0.08 |
| 90 | 4,19 | 0,38 |  |  | 0,10 | 0,01 |  | 0,00 | 6,00 | -186,69 | 387,47 | 4,97 | 0.02 |
| 30 | 4,19 | 0,38 |  | 0,00 | 0,10 | 0,01 |  |  | 6,00 | -186,70 | 387,49 | 4,99 | 0.02 |
| 28 | 4,19 | 0,37 | 0,00 |  | 0,10 | 0,01 |  |  | 6,00 | -186,70 | 387,49 | 4,99 | 0.02 |
| 58 | 4,19 | 0,38 |  |  | 0,10 | 0,01 | 0,00 |  | 6,00 | -186,70 | 387,50 | 5,00 | 0.02 |
| 78 | 4,19 | 0,37 |  | 0,00 | 0,10 |  |  | 0,00 | 6,00 | -186,74 | 387,58 | 5,07 | 0.02 |
| 106 | 4,19 | 0,37 |  |  | 0,10 |  | 0,00 | 0,00 | 6,00 | -186,74 | 387,58 | 5,08 | 0.02 |
| 76 | 4,19 | 0,37 | 0,01 |  | 0,09 |  |  | 0,00 | 6,00 | -186,74 | 387,58 | 5,08 | 0.02 |
| 46 | 4,20 | 0,37 |  | 0,00 | 0,09 |  | 0,00 |  | 6,00 | -186,76 | 387,61 | 5,11 | 0.02 |
| 44 | 4,20 | 0,36 | 0,01 |  | 0,09 |  | 0,00 |  | 6,00 | -186,76 | 387,62 | 5,12 | 0.02 |
| 16 | 4,20 | 0,36 | 0,01 | 0,00 | 0,09 |  |  |  | 6,00 | -186,76 | 387,63 | 5,12 | 0.02 |
| 2 | 4,20 | 0,38 |  |  |  |  |  |  | 3,00 | -191,63 | 389,82 | 7,32 | 0.01 |
| 122 | 4,18 | 0,38 |  |  | 0,10 | 0,01 | 0,01 | 0,00 | 7,00 | -186,68 | 390,22 | 7,72 | 0.01 |
| 94 | 4,19 | 0,38 |  | 0,00 | 0,10 | 0,01 |  | 0,00 | 7,00 | -186,68 | 390,24 | 7,74 | 0.01 |
| 92 | 4,19 | 0,37 | 0,00 |  | 0,10 | 0,01 |  | 0,00 | 7,00 | -186,69 | 390,24 | 7,74 | 0.01 |


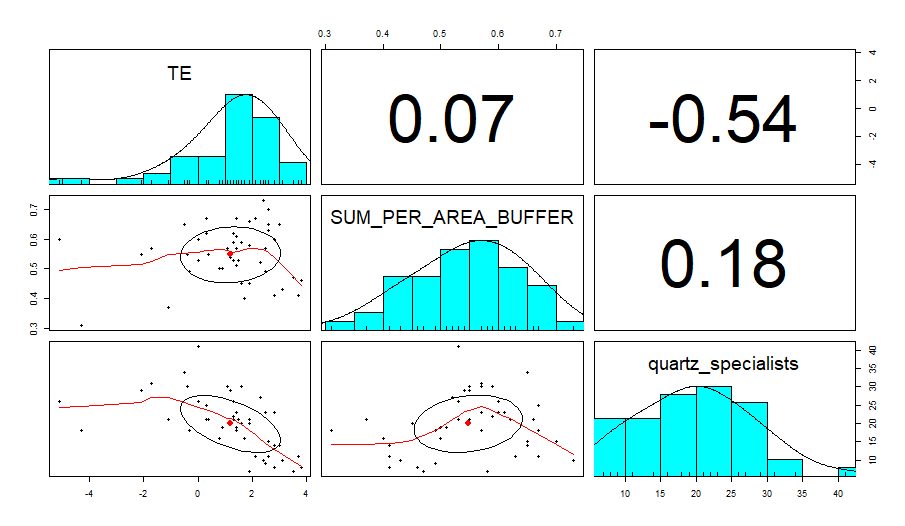


Fig. S5: Pairs-panel figure with the histograms (blue), scatterplots with loess-curves (scatterplots with red line) and the correlation coefficient of the quartz specialist richness (quartz_specialists), the target effect (TE) and the matrix contrast (SUM_PER_AREA_BUFFER). The hump-shaped scatterplot between the quartz specialist richness and the target effect and matrix contrast indicated non-linear relationship between these variables.

Tab S5: Model outputs for single-predictor generalized linear models (glms) for the quartz specialist richness (number of all quartz specialists) and the 5 final predictor variables. We used negative binomial distribution for all models, as most models with Poisson distribution showed overdispersion. To estimate the explained variance of the model (Pseudo-R²), we divided the difference between the null deviance and the residual deviance by the null deviance of the respective model.

|  |  | **Predictor** | **Transformation** | **Index** | **n** | **AIC** | **p-value** | **sign. Level** | **Pseudo-R** |
| --- | --- | --- | --- | --- | --- | --- | --- | --- | --- |
| **Island parameters** |  |  |  |  |  |  |  |  |  |
| Quartz_Rich | ~ | A | log10 | Island area | 47 | 281.76 | <0.001 | *** | 0.64 |
| **Habitat diversity** |  |  |  |  |  |  |  |  |  |
| Quartz_Rich | ~ | DI |  | Habitat diversity index | 47 | 268.43 | <0.001 | *** | 0.73 |
| **Isolation metrics (distance-based)** | | | |  |  |  |  |  |  |
| Quartz_Rich | ~ | DNNI | log10 | Distance to nearest neighbour island | 47 | 326.3 | 0.034 | * | 0.08 |
| **Isolation metrics (area-based)** | | |  |  |  |  |  |  |  |
| Quartz_Rich | ~ | TE |  | Target Effect | 47 | 299.43 | <0.001 | *** | 0.50 |
| **Matrix contrast** |  |  |  |  |  |  |  |  |  |
| Quartz_Rich | ~ | MC |  | Matrix Contrast | 47 | 325.69 | 0.023 | * | 0.13 |

Tab S6: Model outputs by the dredge function for the first 20 multi-predictor generalized linear models (glms) for the quartz species richness and the 5 final predictor variables. We used negative binomial distribution for all models, as most models with Poisson distribution showed overdispersion. The first row (grey) shows the final model.

| **Model number** | **(Intercept)** | **A** | **DI** | **DNNI** | **MC** | **I(MC^2)** | **TE** | **I(TE^2)** | **df** | **logLik** | **AICc** | **delta** | **weight** |
| --- | --- | --- | --- | --- | --- | --- | --- | --- | --- | --- | --- | --- | --- |
| 99 | 2,97 |  | 0,26 |  |  |  | -0,16 | -0,04 | 5 | -127,5 | 266,45 | 0 | 0.15 |
| 35 | 2,93 |  | 0,3 |  |  |  | -0,07 |  | 4 | -129,05 | 267,06 | 0,61 | 0.11 |
| 100 | 2,98 | -0,1 | 0,36 |  |  |  | -0,17 | -0,05 | 6 | -127,04 | 268,19 | 1,73 | 0.06 |
| 107 | 2,97 |  | 0,26 |  | 0,02 |  | -0,17 | -0,04 | 6 | -127,36 | 268,81 | 2,36 | 0.05 |
| 103 | 2,97 |  | 0,25 | -0,02 |  |  | -0,17 | -0,05 | 6 | -127,39 | 268,87 | 2,42 | 0.05 |
| 115 | 2,98 |  | 0,25 |  |  | -0,02 | -0,17 | -0,04 | 6 | -127,39 | 268,89 | 2,44 | 0.05 |
| 3 | 2,93 |  | 0,33 |  |  |  |  |  | 3 | -131,22 | 268,99 | 2,54 | 0.04 |
| 43 | 2,93 |  | 0,29 |  | 0,02 |  | -0,08 |  | 5 | -128,83 | 269,12 | 2,67 | 0.04 |
| 51 | 2,95 |  | 0,28 |  |  | -0,02 | -0,08 |  | 5 | -128,87 | 269,21 | 2,75 | 0.04 |
| 36 | 2,93 | -0,05 | 0,35 |  |  |  | -0,07 |  | 5 | -128,92 | 269,3 | 2,85 | 0.04 |
| 39 | 2,93 |  | 0,3 | 0 |  |  | -0,07 |  | 5 | -129,05 | 269,56 | 3,11 | 0.03 |
| 116 | 3 | -0,12 | 0,36 |  |  | -0,02 | -0,18 | -0,05 | 7 | -126,79 | 270,46 | 4,01 | 0.02 |
| 104 | 2,98 | -0,1 | 0,35 | -0,02 |  |  | -0,18 | -0,05 | 7 | -126,93 | 270,73 | 4,27 | 0.02 |
| 108 | 2,97 | -0,09 | 0,35 |  | 0,01 |  | -0,17 | -0,05 | 7 | -126,98 | 270,83 | 4,38 | 0.02 |
| 67 | 2,92 |  | 0,33 |  |  |  |  | 0,01 | 4 | -131,01 | 270,97 | 4,51 | 0.02 |
| 4 | 2,93 | -0,06 | 0,38 |  |  |  |  |  | 4 | -131,06 | 271,08 | 4,63 | 0.02 |
| 11 | 2,93 |  | 0,32 |  | 0,02 |  |  |  | 4 | -131,13 | 271,21 | 4,75 | 0.01 |
| 111 | 2,97 |  | 0,24 | -0,02 | 0,02 |  | -0,17 | -0,05 | 7 | -127,18 | 271,23 | 4,78 | 0.01 |
| 52 | 2,96 | -0,08 | 0,36 |  |  | -0,03 | -0,08 |  | 6 | -128,62 | 271,34 | 4,88 | 0.01 |
| 119 | 2,99 |  | 0,24 | -0,02 |  | -0,02 | -0,18 | -0,05 | 7 | -127,25 | 271,36 | 4,91 | 0.01 |

Tab S7: Model outputs for single-predictor generalized linear models (glms) for the quartz specialist percentage and the 5 final predictor variables. We used binomial distribution for all models. To estimate the explained variance of the model (Pseudo-R²), we divided the difference between the null deviance and the residual deviance by the null deviance of the respective model.

|  |  | **Predictor** | **Transformation** | **Index** | **n=** | **AIC** | **p-value** | **sign. Level** | **Pseudo-R** |
| --- | --- | --- | --- | --- | --- | --- | --- | --- | --- |
| **Island parameters** |  |  |  |  |  |  |  |  |  |
| Quartz_Perc | ~ | A | log10 | Island area | 47 | 263.26 | 0.07 | n.s. | 0.066 |
| **Habitat diversity** |  |  |  |  |  |  |  |  |  |
| Quartz_Perc | ~ | DI |  | Habitat diversity index | 47 | 265.77 | 0.35 | n.s. | 0.017 |
| **Isolation metrics (distance-based)** | | |  |  |  |  |  |  |  |
| Quartz_Perc | ~ | DNNI | log10 | Distance to nearest neighbour island | 47 | 266.59 | 0.85 | n.s. | 0.0007 |
| **Isolation metrics (area-based)** | | |  |  |  |  |  |  |  |
| Quartz_Perc | ~ | TE |  | Target Effect | 47 | 264.96 | 0.19 | n.s. | 0.03 |
| **Matrix contrast** |  |  |  |  |  |  |  |  |  |
| Quartz_Perc | ~ | MC |  | Matrix Contrast | 47 | 264.27 | 0.12 | n.s. | 0.05 |

Tab S8: Model outputs by the dredge function for the first 20 multi-predictor generalized linear models (glms) for the quartz species percentage and the 5 final predictor variables. We used binomial distribution for all models. The first row shows the final model. The best model according to the dredge output is shown in row 3. Due du a high variance inflation factor, we modified this model and excluded the habitat diversity index (DI) as well non-significant terms (MC). The final model is shown in the first row (grey).

| **Model number** | **(Intercept)** | **A** | **DI** | **DNNI** | **MC** | **TE** | **df** | **logLik** | **AICc** | **delta** | **weight** |
| --- | --- | --- | --- | --- | --- | --- | --- | --- | --- | --- | --- |
| Final_1 | -1,27 | -0,1 |  |  |  | -0,09 | 3 | -127,03 | 260,06 | 3,04 | 0,4 |
| Final_2 | -1,26 | -0,1 |  |  | -0,05 | -0,09 | 4 | -126,33 | 261,61 | 4,59 | 0,24 |
| 28 | -1,27 | -0,4 | 0,34 |  | -0,07 | -0,07 | 5 | -122,78 | 257,02 | 0 | 0.2 |
| 12 | -1,26 | -0,42 | 0,38 |  | -0,08 |  | 4 | -124,04 | 257,03 | 0,01 | 0.2 |
| 20 | -1,27 | -0,37 | 0,29 |  |  | -0,08 | 4 | -124,27 | 257,5 | 0,48 | 0.16 |
| 4 | -1,27 | -0,38 | 0,33 |  |  |  | 3 | -125,89 | 258,33 | 1,31 | 0.1 |
| 16 | -1,26 | -0,42 | 0,38 | 0 | -0,08 |  | 5 | -124,04 | 259,53 | 2,51 | 0.06 |
| 24 | -1,27 | -0,37 | 0,28 | -0,03 |  | -0,08 | 5 | -124,07 | 259,61 | 2,58 | 0.06 |
| 32 | -1,27 | -0,4 | 0,33 | -0,01 | -0,07 | -0,07 | 6 | -122,76 | 259,61 | 2,59 | 0.06 |
| 8 | -1,27 | -0,38 | 0,33 | -0,02 |  |  | 4 | -125,74 | 260,43 | 3,41 | 0.04 |
| 18 | -1,27 | -0,1 |  |  |  | -0,09 | 3 | -127,03 | 260,61 | 3,59 | 0.03 |
| 26 | -1,26 | -0,1 |  |  | -0,05 | -0,09 | 4 | -126,33 | 261,61 | 4,59 | 0.02 |
| 22 | -1,27 | -0,12 |  | -0,04 |  | -0,1 | 4 | -126,6 | 262,15 | 5,13 | 0.02 |
| 2 | -1,26 | -0,07 |  |  |  |  | 2 | -129,63 | 263,53 | 6,51 | 0.01 |
| 30 | -1,26 | -0,11 |  | -0,03 | -0,04 | -0,09 | 5 | -126,09 | 263,65 | 6,63 | 0.01 |
| 10 | -1,26 | -0,06 |  |  | -0,06 |  | 3 | -128,74 | 264,03 | 7,01 | 0.01 |
| 9 | -1,28 |  |  |  | -0,07 |  | 2 | -130,14 | 264,54 | 7,52 | 0 |
| 19 | -1,28 |  | -0,07 |  |  | -0,08 | 3 | -129,03 | 264,61 | 7,59 | 0 |
| 1 | -1,28 |  |  |  |  |  | 1 | -131,31 | 264,72 | 7,7 | 0 |
| 6 | -1,26 | -0,08 |  | -0,03 |  |  | 3 | -129,26 | 265,08 | 8,05 | 0 |
| 25 | -1,29 |  |  |  | -0,07 | -0,05 | 3 | -129,3 | 265,16 | 8,13 | 0 |
| 17 | -1,29 |  |  |  |  | -0,05 | 2 | -130,48 | 265,23 | 8,21 | 0 |
